# Assessing Hepatitis B Knowledge Among Native Hawaiians and Pacific Islanders in San Diego

**DOI:** 10.1007/s10903-021-01236-1

**Published:** 2021-07-13

**Authors:** Siotame Lasitani, Christopher Hattori, Teini Elisara, Maria Rosario Araneta

**Affiliations:** 1 San Diego School of Medicine, University of California, 11381 Zapata Ave. Apt. 24 La Jolla, San Diego, CA 92126 USA; 2 University of California San Diego, La Jolla, San Diego, CA USA

**Keywords:** Hepatitis B, Pacific Islanders, Education, Prevention

## Abstract

**Background:**

Asian-Americans and Pacific Islanders comprise 6% of the U.S. population, but 50% of chronic hepatitis B cases and have a cancer mortality that is 60% higher than non-Hispanic Whites. The objective of this study was to assess knowledge of HBV transmission, symptoms/sequelae and treatment among NHPIs in San Diego.

**Methods:**

Self-administered surveys were distributed using convenience sampling at the 24th Annual Pacific Islander Festival in San Diego in 2018.

**Results:**

Overall knowledge scores were low (mean: 9.8 out of 28) among participants. Compared to U.S.- born (mean: 11.6), participants born in Pacific Island countries and territories (mean: 8.5) had lower knowledge scores (p = 0.017) and lower self-reported vaccination rates (50% vaccinated vs 27%, respectively, p = 0.025).

**Discussion:**

Lack of HBV knowledge, low vaccination rates and the discordance between knowledge and behavior regarding HBV vaccination elicits an urgent need to collaborate with NHPI communities for HBV education, screening, immunization and treatment.

## Introduction

Chronic hepatitis B virus (CHB) infection is a global public health issue affecting nearly 350 million people worldwide. CHB can result in cirrhosis, liver failure and accounts for at least 50% of cases of hepatocellular carcinoma worldwide (1). While an effective hepatitis B (HBV) vaccine has been available since 1982, late introduction and/or limited availability of the vaccine have resulted in low infant immunization and therefore high prevalence of CHB in many regions, particularly Asia and Pacific Island countries and territories (PICT) (2). The HBV prevalence in the United States (U.S.) also reflects this global pattern of HBV endemicity, with the majority of cases being attributed to foreign-born individuals, notably those from Asia and PICT (3). In fact, Asian Americans and Pacific Islanders (AAPIs) contribute to more than 50% of CHB cases in the U.S. (4). This disparity unfortunately translates to a liver cancer death rate that is 60% higher among AAPIs than non-Hispanic Whites despite the availability of effective antiviral treatments to prevent or delay the onset of HBV-related liver disease (3).

Many studies have been done to explore underlying causes for these disparities among highly impacted communities, but have mainly focused on Asian Americans or report data aggregately with Asian Americans and Native Hawaiians and Pacific Islanders (NHPIs) (5–7). In 1997, the Office of Management and Budget implemented a new racial and ethnic category that disaggregated AAPIs into two groups: NHPIs and Asians, and mandated that federal agencies collect and report data using the new racial and ethnic categories by the year 2003. It was recognized that the historically accepted practice of aggregating NHPIs with Asians often mischaracterized NHPIs and obscured the potential elevated risks among NHPIs (8). NHPIs make up only 0.5% of the U.S. population, but are one of the fastest growing populations (9).

Data from the National Cancer Institutes SEER registries showed the age-adjusted incidence rate of liver cancer among Samoan men (30.7 per 100,000) was almost four times higher compared to non-Hispanic White men (8 per 100,000), and twice as high compared to Chinese-Americans (14.5 per 100,000) (10, 11). This raises an urgent need to evaluate NHPI communities to better understand how to promote HBV screening, prevention, and education, particularly for a community where a scarcity of research and data exists (12). This study sought to determine the level of knowledge and awareness of HBV epidemiology, symptoms, transmission, and treatment among NHPI communities in San Diego.

## Methods

### Participants and data collection

San Diego County has the largest NHPI population in the contiguous U.S. (9). Data for this study was collected using a self-administered survey at the 24th Annual Pacific Islander Festival (PIFA) in San Diego in 2018 (13). A convenience sampling approach was used. The co-authors and additional volunteers distributed and collected the surveys during the two-day festival weekend. Surveys were available at a basic health screening and education booth by the University of California, San Diego School of Medicine and widely distributed amongst festival attendees and other craft, food and information vendors/booths. Surveys were only distributed to festival attendees over the age of 18 and who self-identified as NHPI.

The term “foreign-born” has been used in many studies to label populations born in HBV prevalent regions and highlight the higher risk for HBV infection in these communities (5, 6, 14). The U.S. Census Bureau defines “foreign-born” as anyone who is not a U.S. citizen at birth.

However, they recognize anyone born in the U.S., Puerto Rico, a U.S. Island Area (Guam, the Commonwealth of the Northern Mariana Islands, or the U.S. Virgin Islands) as “native born” with American Samoans being considered U.S. Nationals. It is therefore inappropriate to use the term “foreign-born” when including countries from U.S. Island Areas. For the purposes of analysis, “born in a Pacific Island Country or Territory” (PICT-born) instead of “foreign-born” has been adopted to be inclusive of participants born in endemic countries in the Pacific while recognizing the political relationships the U.S. has with U.S. Island Areas (12, 14). “U.S.-born” was used to refer to participants born in one of the 50 states.

### Measures

The self-administered survey consisted of 31 questions and was created based on previously published assessments developed for foreign-born communities at-risk for HBV (Table 2) (5–7). The survey included basic demographic characteristics (age category, race/ethnicity, sex, birthplace) with questions covering HBV epidemiology, transmission and prevention, clinical manifestations and natural history, sequelae and management. Questions about participants’ HBV status, vaccine status and family HBV status were also included. Answer choices for all questions included yes/no/I don’t know (5–7). The survey was reviewed by NHPI community members and key community leaders with minor edits made to improve clarity and to ensure use of culturally appropriate language. English surveys were distributed as most NHPIs speak English and providing surveys in the multitude of languages spoken in this population would be challenging.

### Statistical analysis

Statistical analyses were performed using SAS (Statistical Analysis Systems, Cary, NC). Surveys were excluded from analysis if a participant was not of NHPI descent, listed “Pacific Islander” as race/ethnicity without PICT specification, did not complete all demographic information, or if answers were unclear (i.e. multiple answers for a single question). To evaluate for general knowledge, a single point was assigned for correct answers with no points awarded for incorrect answers, “I don’t know” or left blank. The knowledge score range was 0 to 28 points. The mean for correct answers was calculated to determine the knowledge level of participants. Independent variables included age category, race/ethnicity, sex, birthplace in addition to the last three questions of the survey assessing HBV status, HBV vaccine status and family HBV status. Chi-square analysis tests were conducted to compare HBV knowledge and vaccination status among PICT-born compared to U.S.-born participants. Chi-square analysis was limited to respondents with a complete response. Statistical significance was established as p values <0.05. The anonymous survey used in this study was presented to the University of California San Diego Human Research Protections Program, but was considered exempt from Institutional Review Board approval.

## Results

A total of 132 participants were surveyed anonymously, but only 95 (75.6%) surveys were included in data analysis. The demographic distribution of participants is illustrated in Table 1. Almost three-fourths of the participants identified as either Samoan (41%) or Guamanian/Chamorro (32%), the majority were PICT-born (58%), women (61%), and 75% were younger than age 49 years.

**Table 1:** Demographic characteristics among participants of a self-administered survey on Hepatitis B awareness at the Pacific Islander Festival, San Diego, California, 2018

Main variable	Categories	Frequency (n = 95)	Percent (%)
Race/ethnicity	Carolinian	2	2.1
	Chamorro/Guamanian	30	31.6
	Hawaiian	6	6.3
	Marshallese	9	9.5
	Micronesian	2	2.1
	Palauan	1	1.1
	Samoan	39	41.1
	Tahitian	1	1.1
	Tongan	5	5.3
Country of origin	PICT-Born	55	57.9
	U.S.-Born	40	42.1
Sex	Male	37	38.9
	Female	58	61.1
Age	18–29	30	31.6
	30–39	21	22.1
	40–49	20	21.1
	50–59	14	14.7
	60 +	10	10.5

The average number of correct responses among participants was 9.8 (out of a maximum of 28; range 0–24) with a standard deviation of 6.2. U.S.-born participants had an average of 11.6 (range 0–24) correct responses with a standard deviation of 6.3 and PICT-born respondents had an average of 8.5 (range 0–19) with a standard deviation of 5.9 (p = 0.017). The question with the highest percent correct was “Hepatitis B can be treated with medication” at 65.3% and the lowest percent of correct responses was “Hepatitis B is common in areas with poor sanitation/hygiene” at 8.4% (Table 2). For many questions, “I don’t know” was a common answer with “Hepatitis B can cause cancer” resulting with the highest percent of uncertainty at 51.6%. Survey respondents had mixed knowledge of the chronicity (45.7%) and asymptomatic nature of HBV (21.3%), and availability of treatment for (65.3%), and sequelae of CHB (19.8%) (Table 2).

**Table 2:** Survey questions and response percents among participants of a self-administered survey on Hepatitis B awareness the Pacific Islander Festival, San Diego, California, 2018

Question	Yes	No	Don’t Know
Symptoms of hepatitis B may include:			
Jaundice (yellow skin, eyes)	43.2	17.9	37.9
Blurry vision	31.6	22.1	42.1
Abdominal pain (stomach pain)	30.5	20	42.1
No symptom at all	17.9	31.6	34.7
Hepatitis B can be transmitted by:			
Coughing/sneezing	30.5	29.5	34.7
Sharing needles (drugs, etc.)	53.7	13.7	30.5
Mother to child at birth	44.2	15.8	33.7
Contaminated water or food (poor sanitation)	47.4	21.1	28.4
Stress	7.4	47.4	38.9
A virus	36.8	22.1	37.9
Through sex	36.8	22.1	36.8
Exposure to infected blood	57.9	11.6	26.3
Hepatitis B:			
Can cause cancer	19	25.3	51.6
Can cause symptoms in most people	35.8	14.7	43.2
May not cause symptoms in most people	26.3	21.1	49.5
Always goes away on its own within a few weeks	5.3	44.2	43.2
Mainly affects the heart	11.6	28.4	50.5
Mainly affects the liver	40	12.6	44.2
Can remain in the body for life	44.2	13.7	38.9
Can be spread to family members and other close contacts	43.2	10.5	41.1
Is caused by drinking too much alcohol	11.6	35.8	46.3
Hepatitis B is common:			
Amongst Pacific Islanders	32.6	18.9	44.2
In areas with poor sanitation/hygiene	55.8	8.4	32.6
If you or your parents have immigrated from a country	29.5	18.9	47.4
Where the virus is endemic (Asia, Africa, etc.)			
Hepatitis B is preventable by:			
Washing hands	48.4	19	28.4
A vaccine	62.1	11.6	25.3
Hepatitis B can be:			
Treated with medications	65.3	8.4	26.3
Controlled with regular exercise	14.7	30.5	48.4
I have been vaccinated against hepatitis B	36.8	28.4	31.6
I have hepatitis B	5.3	67.4	23.2
I have a family member with hepatitis B	10.5	48.4	36.8

*Questions do not add up to 100% as percent left blank is not included in table

When stratified by nativity, there were no differences in knowledge of symptoms and sequelae of HBV. Knowledge about transmission were similar by nativity, with the exception of needle sharing and the etiology of HBV; only 44% of PICT-born responses knew that HBV could be transmitted by needle sharing compared to 69% of U.S.-born (p = 0.018). 58% U.S.-born respondents knew that HBV could be spread to family members and other close contacts compared to 37% of PICT-born (p = 0.047). Only one of every four (24%) PICT-born respondents knew that HBV is caused by a virus, compared to over half (58%) of U.S.-born participants (p < 0.001). Almost three of every four (74%) PICT-born respondents incorrectly identified alcohol overuse as a cause of HBV compared to less than half (43%) of U.S.-born (p < 0.003).

When asked about HBV vaccination, 62.1% of participants indicated awareness of a vaccine. Of these participants, 52.6% indicated they had been vaccinated. Compared to U.S.-born participants PICT-born participants, 22.5 % vs. 38%, did not know their vaccination status, 50% vs. 27% were vaccinated (p = 0.025) and 25% vs. 31% did not receive a vaccine, respectively. When asked about their HBV status, 5% of participants indicated they had HBV, 20% being PICT-born. When asked about family member HBV status, 10.5% of respondents indicated they had a family member with HBV, half of whom were PICT-born (p > 0.05). Of participants who indicated they had HBV, 60% also indicated they had a family member with HBV.

## Discussion

Similar to other studies assessing HBV knowledge in high risk populations, “I don’t know” was the most common response overall and knowledge scores were low, with less than half of questions answered correctly, reflecting a general lack of knowledge about HBV (5). The lack of knowledge of transmission, chronicity, sequelae, and treatment of CHB, in addition to the high level of uncertainty regarding hepatocellular carcinoma as a sequela of CHB are particularly concerning within the context of the disparate rates of liver cancer death among AAPIs (3). It is of utmost importance that future educational interventions focus on these areas particularly in a high risk population demonstrating a high percent of uncertainty regarding their HBV disease burden (16). Other areas of focus include education about the different hepatitides and their symptoms, transmission and treatment as confusion is common. One study measuring HBV transmission knowledge and self-reported screening/testing behavior among Pacific Islanders in Southern California reported respondent confusion between hepatitis A and B transmission (16). Similarly, in this study the question with the lowest percent of correct responses was “Hepatitis B is common in areas with poor sanitation/hygiene”.

Given that the majority of HBV cases in the U.S. are attributed to foreign-born individuals from Asia and PICT, this study sought to analyze differences in HBV knowledge and vaccination status between U.S.-born and PICT-born respondents. Other factors (i.e. medical coverage, employment status, knowledge, and information source) and their relation to HBV screening and vaccination behavior among NHPIs have been explored, but immigration status has yet to be explored (16). Most alarming, self-reported vaccination rates were significantly lower among PICT-born respondents compared to U.S.-born and more PICT-born respondents were unaware of their HBV vaccine status compared to U.S.-born. Other significant differences of HBV knowledge between PICT-born and U.S.-born respondents included etiology, most notably that HBV is a virus, and transmission to family members and close contacts and by sharing needles. These findings highlight the urgent need to partner with NHPI communities to provide culturally appropriate health/education interventions to improve screening and immunization among PICT-born individuals.

Of note, among the participants that indicated awareness of a vaccine for HBV prevention, only about half reported having been vaccinated, suggesting a discordance between knowledge and behavior. Of the participants who indicated having HBV, a majority of them also indicated having a family member with HBV. NHPIs have the highest percentage of multigenerational family households, raising a unique concern of horizontal HBV transmission among these communities (17). While horizontal transmission is higher in Pacific Island countries and territories than in the U.S., a multigenerational approach for health and educational interventions is likely to be more effective in NHPI communities (18). A comprehensive strategy could also include working with primary care clinicians to raise awareness about PICT-born individuals coming from HBV endemic regions, who could benefit from screening and follow-up with monitoring and treatment (6).

While there are many important takeaways from this study, this study did have some limitations. Because this was a convenience sample, the results must be viewed as suggestive rather than conclusive. The surveys were also administered at one location in San Diego. Distributing surveys at churches, halaus, Pacific Islander markets, etc. would not only have increased our sample size but may have been more representative of the NHPI community in San Diego. Therefore, our findings should only be considered for the participants of this study and not generalized to all NHPI communities in San Diego. Also, even though a majority of NHPIs speak English and could participate in our survey, we were not able to include non-English speakers. Non-English speakers are more likely to be PICT-born and therefore be at risk of CHB and should be taken into consideration when strategizing health and educational interventions. The survey did not elicit information regarding access to health care, years of education, nor the number of years PICT-born participants resided in the US – variables which might have informed the differences in responses by nativity. Lastly, as with all self-administered surveys, reported HBV status, HBV vaccine status and family HBV status were not validated against medical records and may not be accurate.

## Conclusion

Our findings demonstrate that there is a general lack of knowledge of the transmission, chronicity, sequelae and treatment of CHB among Pacific Islanders. The low vaccination rates amongst all participants, particularly among PICT-born, and the discordance between knowledge and behavior regarding HBV vaccination elicits urgent efforts for partnering with NHPI communities to provide HBV education, screening, immunization and treatment.

## References

[1] Xie Y, Hepatitis B virus-associated hepatocellular carcinoma. In: Cai Q, Yuan Z, Lan K, editors. Infectious agents associated cancers: epidemiology and molecular biology advances in experimental medicine and biology. Springer: Singapore 2017 p.11–21

[2] Hepatitis B. https://www.who.int/news-room/factsheets/detail/hepatitis-b Accessed Oct 27 2020

[3] CDC. People Born Outside of the United States and Viral Hepatitis. Published September 24, 2020. https://www.cdc.gov/hepatitis/populations/Born-OutsideUnited-States.htm. Accessed Nov 3 2020

[4] NIDDK. Hepatitis B. National Institute of Diabetes and Digestive and Kidney Diseases. . https://www.niddk.nih.gov/health-information/liver-disease/viralhepatitis/hepatitis-b Accessed Oct 19 2020

[5] Bride M (2018). Assessing hepatitis B knowledge among immigrant communities in New York City. J Immigr Minority Health.

[6] Shiau R (2012). Using survey results regarding hepatitis B knowledge, community awareness and testing behavior among Asians to improve the San Francisco hep B free campaign. J Commun Health.

[7] Ma GX (2007). Knowledge, attitudes, and behaviors of hepatitis B screening and vaccination and liver cancer risks among Vietnamese Americans. J Health Care Poor Underserved.

[8] Panapasa SV, et al: Efficacy of Federal Data: Revised Office of Management and Budget Standard for Native Hawaiian and Other Pacific Islanders Examined. AAPI Nexus Journal: Policy, Practice, and Community. 2011;9((1,2)):212–220. 10.17953/appc.9.1-2.cp21x04488016643 PMC4211287 25360070

[9] US Census Bureau. The Native Hawaiian and Other Pacific Islander Population: 2010. The United States Census Bureau. https://www.census.gov/library/publications/2012/dec/c2010br-12.html Accessed Nov 3 2020

[10] Liu L (2013). Cancer incidence trends among native Hawaiians and other Pacific Islanders in the United States, 1990–2008. J Natl Cancer Inst.

[11] Thompson CA (2016). The burden of cancer in Asian Americans: a report of national mortality trends by Asian ethnicity. Cancer Epidemiol Biomarkers Prev.

[12] Howell J (2014). An overview of hepatitis B prevalence, prevention, and management in the Pacific Islands and Territories. J Gastroenterol Hepatol.

[13] Pacific Islander Festival Association San Diego. http://www.pifasandiego.com/ Accessed Nov 3 2020

[14] Kowdley KV (2012). Prevalence of chronic hepatitis B among foreign-born persons living in the United States by country of origin. Hepatology.

[15] US Census Bureau. About the Foreign-Born Population. The United States Census Bureau. 2020. https://www.census.gov/topics/population/foreign-born/about.html Accessed Nov 3

[16] Takahasi LM (2011). Hepatitis B among Pacific Islanders in Southern California: How is Health information associated with screening and vaccination?. J Community Health.

[17] US Census Bureau. Multigenerational Households: 2009–2011. The United States Census Bureau. https://www.census.gov/library/publications/2012/acs/acsbr11-03.html Accessed Nov 3 2020

[18] Danielsson N (2009). Improved immunization practices reduce childhood hepatitis B infection in Tonga. Vaccine.

